# Development and Characterization of 20 Microsatellite Markers for Chinese Black Sleeper, *Bostrychus sinensis*

**DOI:** 10.3390/ijms120x000x

**Published:** 2011-12-20

**Authors:** Hang-Jun Wang, Hung-Du Lin, Li-Yan Zhang, Shao-Xiong Ding

**Affiliations:** 1 State key labratory of Marine Environmental Science, Xiamen University, Xiamen 361005, China; E-Mail: 22420091151159@stu.xmu.edu.cn; 2 The laboratory of Marine Biodiversity and Global Change, Xiamen University, Xiamen 361005, China; 3 Department of Oceanography, Xiamen University, Xiamen 361005, China; E-Mail: summer_0917@163.com; 4 Department of Physical Therapy, Shu Zen College of Medicine and Management, Kaohsiung 821, Taiwan; E-Mail: varicorhinus@hotmail.com; 5 Fujian institute of oceanography, Xiamen 361005, China

**Keywords:** *Bostrychus sinensis*, microsatellites, population genetic

## Abstract

Twenty microsatellite markers were isolated and characterized from the Chinese black sleeper, *Bostrychus sinensis*. Loci were screened in 30 individuals from Taiwan. For each locus, the number of alleles varied from 4 to 22 with mean expected and observed heterozygosity of 0.79 and 0.66, respectively. One locus significantly deviated from Hardy-Weinberg equilibrium after Bonferroni correction and no significant linkage disequilibrium was detected. This set of microsatellites will provide a suitable tool for population genetic studies of Chinese black sleeper.

## 1. Introduction

The Chinese black sleeper, *Bostrychus sinensis* (Lacepede 1801), is one of the most widespread species of Indo-Pacific eleotrids, manifesting on the northern Indian Ocean coast, reaching east to the Pacific, Melanesia and Polynesia, north to Japan, and south to Australia [1,2]. In China, it is distributed in the coastal area of the East China Sea, Taiwan Strait and South China Sea. Chinese black sleepers are burrowing amphibians. They live in a limited territory, spawn in their burrow, and exhibit egg-guarding behavior. These factors suggest that populations will be sensitive to local environmental conditions and have a low rate of dispersal. Therefore, *Bostrychus sinensis* can be a useful biological indicator of the effects of long-term historical vicariant events and short-term human activities on intertidal habitats. In order to facilitate its population genetic studies, we developed and characterized 20 polymorphic microsatellite markers from Chinese black sleeper.

## 2. Results and Discussion

The number of observed alleles per locus ranged from 4 to 22. The observed and expected heterozygosity values ranged from 0.200 to 0.889 and 0.186 to 0.933, respectively. One locus (S98W83) significantly deviated from Hardy-Weinberg equilibrium after Bonferroi correction (*P* < 0.0025) and no significant genotypic linkage disequilibrium (LD) was found between all pairs of these 20 loci after Bonferroni correction (*P* > 0.0025). The levels of polymorphism uncovered at these loci suggest that they should be useful for population genetics as well as phylogeographic studies.

## 3. Experimental Section

### 3.1. Isolation of Microsatellite Markers

Microsatellites from *B.sinensis* were isolated using a modified enrichment technique described by Ding [3]. Genomic DNA was extracted from the muscle tissue using a standard traditional phenol-chloroform procedure [4] and digested with *Sau*3AI at 37 °C overnight. Fragments from 500–2000 bp were excised from agarose gels using a QIAqucik Gel Extraction Kit (QIAGEN) and ligated to two oligo adapters (Oligo A 5′-GATCGTCGACGGTACCGAATTCT-3′ and Oligo B 5′-GTCAAGAATTCGGTACCGTCGAC-3′) to facilitate amplification by PCR. The amplified genomic fragments were subsequently hybridized with the 5′-biotinylated oligo probes ATA(CA)22C and fragments containing potential repeat motifs were captured with steptavidin-coated magnetic beads (Dynabeads^®^ M-280, Invitrogen). To increase the amount of potential repeat motifs, a “recovery” PCR was performed using oligo B as the PCR primer. The PCR products were then purified and ligated into PMD19-T vector (TAKARA) and transformed into DH5α competent cells. Cells were then plated onto LB agar, X-gal and ampicillin and incubated overnight at 37 °C.

The positive clones were identified by PCR with vector-specific primers. After being identified, 196 positive clones were randomly selected for sequencing using ABI3730XL sequencer (Applied Biosystems). Chromatograms were assembled and edited using SEQUENCHER 4.9 (Gene Codes Corporation), and 70 primers were designed for each unique amplicon containing a target microsatellite repeat. Initially, eight samples from different localities were used to test amplification of loci and evaluate polymorphic content. The PCR amplification was performed in 15 μL volume containing 10 mM Tris-HCl (pH 8.3), 50 mM KCl, 1.5 mM MgCl_2_ 0.2 mM each dNTPs, 0.4 μM each primer, 1 U *Taq* polymerase (TAKARA) and 30 ng genomic DNA. After denaturation for 5 min at 94 °C, followed by amplication for 30 cycles (94 °C for 30 s, annealing temperature for each pair of primers (Table 1) for 30 s, 72 °C for 30 s) and a final step at 72 °C for 5 min. PCR products were mixed with the GS500LIZ size standard (Applied Biosystems) and formamide and run on an ABI3130xl DNA Analyzer. Fragment analysis and genotyping were performed using Genemapper version 4.0 (Applied Biosystems). Out of the 70 primer pairs tested, 20 pairs were successfully amplified by PCR and further characterized using additional samples at Chiku Lagoon (23°55′05″ N-120°02′57″ E) from Taiwan (*n* = 30).

### 3.2. Data Analysis

The number of alleles, observed and expected heterozygosities, *P* value of Hardy-Weinberg and linkage disequilibria were estimated by using POPGENE [5].

## 4. Conclusions

In the present study, we describe 20 polymorphic microsatellite loci shown as the first set of microsatellite markers designed specifically for *Bostrychus sinensis.* These loci would be useful in providing an effective tool for investigating genetic variation and population structure in *B. sinensis*. Microsatellites are an excellent choice of genetic marker for genome mapping due to their hyper-variability and abundance throughout most vertebrate genomes. In our results, there are some strong heterozygosities for microsatellite loci, like BSD137/BSW045/BSW053/BSC001, which are suitable for identifying genetic mapping in *B.sinensis*. These markers will prove helpful in the management of fisheries and in the design of conservation strategies.

## Figures and Tables

**Table 1:** Details for 20 polymorphic microsatellite loci developed for *Bostrychus sinensis*.

Locus Genbank no.	Repeat motif	Primer sequence(5′-3′)	*T*_a_ (°C)	Size range (bp)	*N*a	*H*_o_/*H*_e_	*P*-value

BSD026	(CA)_61_	F: CATAAAAGACCCATTGTAACTGCT	58	256–280	8	0.865/0.825	0.0505
JN806116		R: CTGTAGCCCTCAGGAGCACATA					
BSD121	(AC)_19_	F: CGCACTGTCATCATAGCACTC	58	131–175	8	0.444/0.760	0.0060
JN806117		R: CCACCTGACAATGATTTAGTT					
BSD137	(TG)_23_	F: CTGACCTGGACTTCCCCTGG	58	202–330	22	0.879/0.933	1.0000
JN806118		R: CTGGGACAGGAGATGAGTTTT					
BSB006	(TG)_6_C(GT)_13_	F: TATTCTGTAATTACTGATATGTGCA	60	172–222	9	0.742/0.843	0.2209
JN806119		R: TACACAAGACCAAAAAGGTTAGGAA					
BSW045	(CT)_6_…(AC)_70_	F: ACTTTTTTCTCAATTTGGTTTCTAA	52	128–220	14	0.833/0.867	0.9975
JN806120		R: TGTGCTCAGGGGTACCGGGA					
BSW068	(AC)_16_	F: CTACAACAGCATCAGCCAACC	58	113–133	7	0.684/0.766	0.3483
JN806121		R: ACTCCCAAACACTGTCCAAGAAC					
BSSD14	(TG)_43_	F: ATTTAGCGAGGCTTTATGTT	55	200–244	11	0.567/0.754	0.5198
JN806122		R: GGCTGGCTTCCATCTTTTCT					
BSSD21	(TG)_38_	F: GATCCATTCTTAAAACACTCGTTAT	55	267–313	9	0.774/0.833	0.6818
JN806123		R: CAGGAGCAGTATCCAGACAAAA					
BSSW83	(TG)_16_	F: CCAGCAGCACCTGACACTCCAT	58	144–156	8	0.452/0.808	0.0000 *
JN806124		R: TCCAGTGTTTGAAACTCCTGCC					
BSE020	(GT)_25_	F: GATTTTCAGAGCAGCAGCGTTGGC	66	239–339	10	0.769/0.824	0.9217
JN806125		R: CCACAAACGGAGCGTCCCAAATCT					
BSSW87	(GT)_35_	F: CGCACAGTTGACGCTTCCTTTA	64	314–388	10	0.680/0.860	0.7399
JN806126		R: GCCTCCCTGTCAGCCTTCACT					
BSSW89	(GT)_33_	F: TTGTAGCATTCCTTCTGCCTGT	52	182–246	10	0.583/0.884	0.1589
JN806127		R: CTCACTCCATCGGAATGTGTCTA					
BSD106	(CA)_20_T(AC)_9_	F: GAGATGAGCAACAGGTGAGTC	56	338–388	10	0.667/0.842	0.5932
JN806128		R: CTGGCAGAAGAGGATTGATGG					
BSD045	(GT)_35_	F: AAATGGATGTGTGAGAATGTGAGGCA	62	258–384	10	0.467/0.720	0.4404
JN806129		R: TGTGAACTCGAATGTGGGAGGTACT					
BSW115	(GT)_37_C(TG)_17_	F: TGTGATGTGTGTTTTGGGTGGTTA	64	437–541	7	0.708/0.764	0.8613
JN806130		R: TGTGTCCTCTGAAGTACCTGAAGC					
BSW053	(AC)_5_… (CA)_7_… (AC)_50_	F: TGCCCCCCAGATACCGACATTA	66	308–394	17	0.889/0.912	0.9997
JN806131		R: CGAGAGGTGAGCCAGGTTCAGGACT					
BSD125	(CA)_6_CGCACG(CA)_35_	F: CGCTTCAGTTCTGTGGAGGTA	56	137–185	9	0.714/0.827	0.9570
JN806132		R: CTGTCTGCCAAAGTTCCTGTTA					
BSC001	(GT)_48_	F: CTTGTTATGTCAAACCGTAGCCTTA	56	341–401	15	0.615/0.909	0.4762
JN806133		R: CCCTATCGTCCCCTGTAGACCG					
BSE008	(TG)_19_	F: GCTGCTCATAAACAATCACTTC	58	138–154	6	0.656/0.734	0.7305
JN806134		R:GTTGTCTGTAATCAGTGGCTCTA					
BSC002	(ACT)_18_	F: ATCAGCATCACAATGACCTGGGAG	55	247–271	4	0.200/0.186	0.9926
JN806135		R: CTTGGTGGAACTACAGACTTTTACA					

*T*_a_ Annealing temperature (°C), *N*a Number of alleles, *H*_o_/*H*_e_ Observed heterozygosity and Expected heterozygosity, *P*-value, *P*-values for exact tests for Hardy-Weinberg equilibrium(HWE),

* Show significant deviation from HWE after Bonferroni correction (*P* < 0.0025)
